# Treatment of Trauma Victims: Consideration of the Whole Perspective

**DOI:** 10.5812/traumamon.5249

**Published:** 2012-07-31

**Authors:** Ali Ebrahimi

**Affiliations:** 1 Section Editor, Head, Trauma Research Center, Baqiyatallah University of Medical Sciences, Tehran, IR Iran

**Keywords:** Trauma, Treatment

As trauma surgeons, we are commonly engaged in treating injuries caused by impacts. For many years, advancements in the care and management of the injured were strongly linked to military conflicts (1). Injury is the number one public health problem in the USA, with a price tag of over $260 billion annually (2). In Iran, road accidents are still the most common cause of mortality among civilians (according to annual reports by police officials).

Military actions in Iraq and Afghanistan and more recently in some Arab countries have resulted in a number of important changes on how trauma victims are managed. We must continually ask ourselves: What can we do to better manage and reduce the number of trauma victims and what can others do to help? A multitude of factors must be considered by the government regarding trauma prevention, management and research. Our duty is to extend training of trauma surgeons, trauma teams, and task forces and to upgrade trauma centers with modern state-of-the art medical equipment. In addition to general surgery residency as the minimal requirement for operating as a trauma surgeon, we must train fellows of trauma and critical care. Trauma fellowships are designed to teach the trainee the various components of triage, emergency care and to run an organized trauma center (3). At present, training of trauma surgeons for general hospitals as well as for trauma centers and preparing them for mass casualties and national disasters is underway; this is necessary to decrease the high mortality of accidents (traffic, earthquake, floods, and conflicts) in our country. Additionally, we need active committees in trauma centers to develop and publish a comprehensive statewide trauma system and disaster protocol; these committees must be coordinated with emergency medical services and trauma research centers nationwide and should have a comprehensive protocol defining responsibilities in disaster management. Modern diagnostic and therapeutic facilities to expedite the management of victims and a scientific database for documentation and research are basic necessities to better manage trauma patients in the future. Thus, the current view point and overall perspective remains to be a comprehensive and holistic one integrating governmental and nongovernmental organizations, surgeons and trauma centers in planning, preparing for and managing mass casualties.
